# Research on the Mechanism of Kaempferol for Treating Senile Osteoporosis by Network Pharmacology and Molecular Docking

**DOI:** 10.1155/2022/6741995

**Published:** 2022-02-03

**Authors:** Fuyu Tang, Peng Zhang, Wenhua Zhao, Guangye Zhu, Gengyang Shen, Honglin Chen, Xiang Yu, Zhida Zhang, Qi Shang, De Liang, Xiaobing Jiang, Hui Ren

**Affiliations:** ^1^Guangzhou University of Chinese Medicine, Guangzhou 510405, China; ^2^Liuzhou Hospital of Chinese Medicine (Liuzhou Hospital of Zhuang Medicine), Guangxi Zhuang Autonomous Region, Liuzhou, 545000, China; ^3^Lingnan Medical Research Center of Guangzhou University of Chinese Medicine, Guangzhou 510405, China; ^4^The First Affiliated Hospital of Guangzhou University of Chinese Medicine, Guangzhou 510405, China

## Abstract

Kaempferol (KP), as a natural anti-inflammatory compound, has been reported to have curative effects on alleviating senile osteoporosis (SOP), which is an inflammation-related musculoskeletal disease, but the molecular mechanisms remain unclear due to scanty relevant studies. We predicted the targets of KP and SOP, and the common targets of them were subsequently used to carry out PPI analysis. Moreover, we adopted GO and KEGG enrichment analysis and molecular docking to explore potential mechanisms of KP against SOP. There were totally 152 KP-related targets and 978 SOP-related targets, and their overlapped targets comprised 68 intersection targets. GO enrichment analysis showed 1529 biological processes (*p* < 0.05), which involved regulation of inflammatory response, oxidative stress, regulation of bone resorption and remodeling, osteoblast and osteoclast differentiation, etc. Moreover, KEGG analysis revealed 146 items including 44 signaling pathways (*p* < 0.05), which were closely linked to TNF, IL-17, NF-kappa B, PI3K-Akt, MAPK, estrogen, p53, prolactin, VEGF, and HIF-1 signaling pathways. By means of molecular docking, we found that kaempferol is bound with the key targets' active pockets through some connections such as hydrogen bond, pi-alkyl, pi-sigma, pi-pi Stacked, pi-pi T-shaped, and van der Waals, illustrating that kaempferol has close combination with the key targets. Collectively, various targets and pathways involve in the process of kaempferol treatment against SOP through regulating inflammatory response, oxidative stress, bone homeostasis, etc. Moreover, our study first reported that kaempferol may regulate core targets' expression with involvement of inflammatory response, oxidative stress, and bone homeostasis, thus treating SOP.

## 1. Introduction

Senile osteoporosis (SOP) is an inflammation-related musculoskeletal disease with serious complications including spine deformation, osteoporotic fracture, and bone pain [1, 2]. Osteoporotic vertebral fracture (OVF) is the worst-affected complication in SOP patients with about 1.8 million vertebral fractures estimated happening every year in China, and the number of vertebral fractures is predicted to increase to 3 million in 2050 [3]. SOP poses serious threats to senior citizens' life and health, which adds to social and family burdens. The treatment of SOP involves the use of drugs inhibiting bone resumption clinically, but long-term use of these drugs can result in some complications, which limit their clinical application [4]. Recently, more and more scholars attach increasing attention to the osteoprotective effect of traditional Chinese medicine on treating SOP [5].

Kaempferol (KP, PubChem CID: 5280863) is a flavonoid identified in various natural products and traditional Chinese medicine like *Drynariae Rhizoma* [6]. KP has been reported to have the curative effect of treating SOP by acting on both osteoblasts and osteoclasts, which may exert osteogenic and antiosteoclastic effects [7]. The current study has illustrated that KP could influence adipogenesis [8], inflammation [9], oxidative stress [10], osteoblastic apoptosis [11], and osteoclastic apoptosis [12], resulting in osteoprotective effects. Therefore, KP could serve as a complementary and alternative medicine with a good prospect for clinical application on treating SOP.

In our prevent study, we performed bioinformatics analysis including network pharmacology and molecular docking so as to carry out systematic analysis on numerous pathways and targets involved in the function of KP on treating SOP.

## 2. Materials and Methods

**Figure 1:**
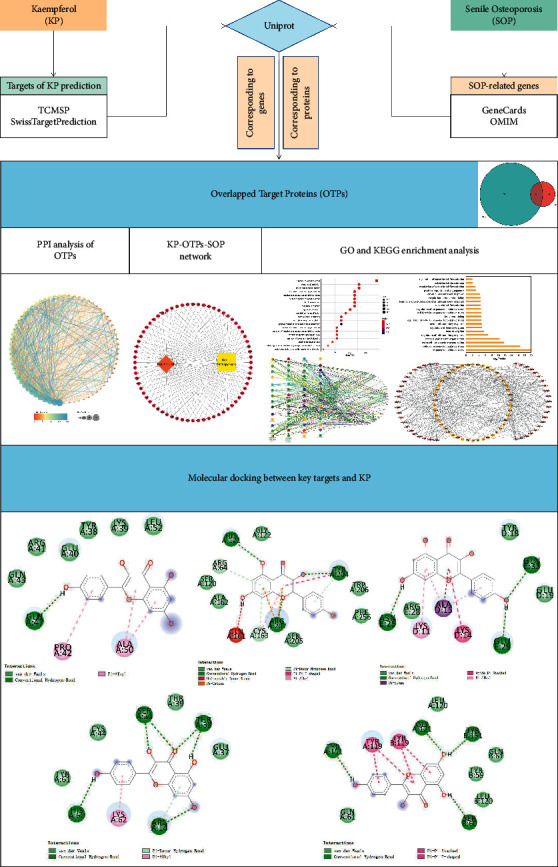
The flowchart of this study.

Figure 1 describes the flow chart of study design.

### 2.1. Obtaining KP-Related Structure and Targets

We obtained the KP-related structure and targets through the following steps: first, we conducted data retrieval on the TCMSP database (https://tcmsp-e.com/) [13], which provides comprehensive information of KP including its structure and target information; second, by searching the PubChem database (https://pubchem.ncbi.nlm.nih.gov/), the KP structure was stored as an “SDF” file, which was imported into the SwissTargetPrediction database (http://new.swisstargetprediction.ch/) [14] to get the targets associated with KP; and third, we adopted the UniProt database (http://www.uniprot.org/uniprot/) to standardize the KP-related target proteins with “popular organisms” limited to humans, which were described as gene symbols.

### 2.2. SOP-Related Genes and Corresponding Proteins

The key word “Senile Osteoporosis” was searched in the two databases, including GeneCards (https://www.genecards.org/) [15] and Online Mendelian Inheritance in Man (OMIM, https://omim.org/) [16], with the species set as “*Homo sapiens*.” The UniProt database was adopted to standardize the corresponding proteins of SOP-related genes.

### 2.3. Overlapped Target Proteins (OTPs)

R (v3.6.1) software was used to take the overlap of KP- and SOP-related target proteins to get OTPs.

### 2.4. Protein Interaction Analysis of OTPs

The STRING database (https://string-db.org/) [17] was retrieved to get the protein-protein interaction (PPI) data of OTPs. Next, the PPI information of OTPs was input into Cytoscape (v3.7.2) software (https://www.cytoscape.org/) [18] to construct the PPI network and calculate the degrees of targets in the network through network topology analysis. We determined the target proteins with degree above average to be core target proteins. Afterwards, we generated a KP-OTPs-SOP network via Cytoscape.

### 2.5. GO Enrichment Analysis and KEGG Pathway Analysis

We conducted GO and KEGG analysis of the overlapped targets by means of clusterProfiler package (R3.6.1) and extracted the enrichment results with *p* < 0.05.

### 2.6. Molecular Docking between Key Targets and KP

The top 5 proteins in terms of degree were chosen for molecular docking, which were considered the key targets in the process of KP treating SOP. In order to explore interaction activity between KP and its key targets, we utilized AutoDock Vina (v1.1.2) software [19] to carry out molecular docking simulations. We searched the PubChem database (https://pubchem.ncbi.nlm.nih.gov/) for the 3D structure of KP. We used AutoDock Tools (v1.5.6) to distribute charge and combine nonpolar hydrogen for KP and converted the results into a PDBQT file. We downloaded the crystal structures of target proteins from the RCSB PDB website (https://www.rcsb.org/). Then, the target protein was separated from its ligand, added polar hydrogen, and distributed charge via AutoDock Tools, which would be subsequently stored as a PDBQT file. AutoDock Tools were also utilized to calculate the center and size of the docking box. Molecular docking simulations among KP and the target proteins were performed with every affinity calculated. Afterwards, Discovery Studio (https://www.3ds.com/products-services/biovia/products/molecular-modeling-simulation/biovia-discovery-studio/) was used to draw and analyze the docking results of KP.

## 3. Results

### 3.1. KP-Related Structure and Target Proteins

From TCMSP and SwissTargetPrediction databases, we got 152 targets of KP. With them imported into the UniProt database, we obtained KP-related target proteins called gene symbols. Supplementary Tables S1 and S2 show the KP-related structure and target information.

### 3.2. Target Information of SOP and Overlapped Target Proteins (OTPs)

**Figure 2:**
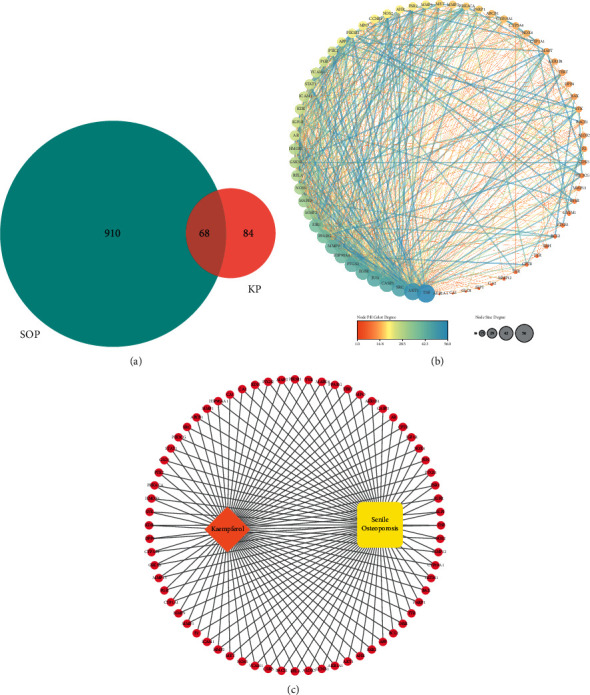
Venn diagram of OTPs (a), PPI network of OTPs (b), and KP-OTPs-SOP network (c).

Through the retrieval of GeneCards and OMIM databases, we obtained a total of 978 target proteins of SOP. We took the overlap of KP- and SOP-related targets as OTPs, which included 68 overlapped targets, as demonstrated in Table 1 and Figure 2(a).

### 3.3. PPI Network Construction and Core Target Protein Screening

OTPs were imported into the STRING database with the targets having no interactive connections with others hidden. And then we imported the PPI data into Cytoscape (v 3.7.2) to draw PPI network in Figure 2(b). There were 28 target proteins predicted to be the core target proteins (Table 2), whose degrees were above average degree (20.59).

### 3.4. KP-OTPs-SOP Network Plotting

Figure 2(c) shows the KP-OTPs-SOP network with 70 nodes and 136 edges included. In Figure 2(c), the red circular nodes stand for the overlapped target proteins (OTPs). The orange diamond node stands for “kaempferol.” The yellow round rectangle node stands for “senile osteoporosis.” The edges stand for the interactive relationships among kaempferol, senile osteoporosis, and the overlapped targets.

### 3.5. GO Enrichment Analysis

**Figure 3:**
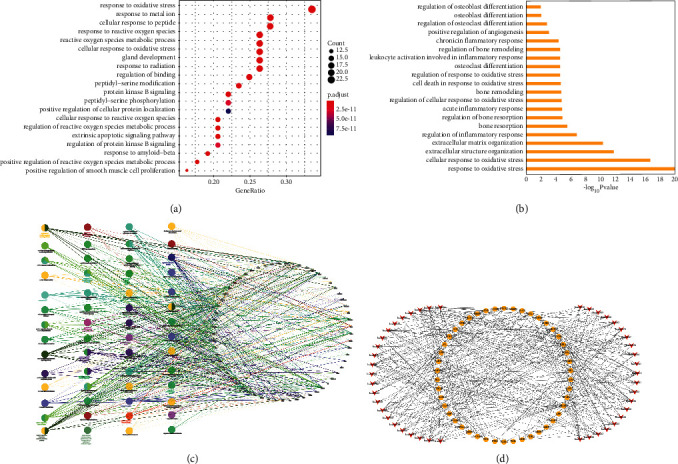
GO.BP enrichment analysis (a–c) and pathway-target network (d). (a, b) The top and screened 20 items of biological processes in terms of *p* value. (c) Different colors represent different biological process groups, and node size stands for term *p* value, while the edges represent the connections between biological processes and targets. (d) A red V-shaped node represents a signaling pathway, a yellow circular node represents a gene, and an edge represents a relationship between a pathway and a gene.

We got 1529 items of biological process (BP). The top 20 items are shown in Figure 3(a). Noteworthily, we have filtrated 20 entries mainly related to inflammatory response, oxidative stress, angiogenesis, bone remodeling and resorption, and osteoblast and osteoclast differentiation, which have a close association with bone homeostasis as demonstrated in Figure 3(b). Additionally, we input 68 OTPs into Cytoscape for GO.BP enrichment analysis with *p* value set to 0.00001. Figure 3(c) illustrates the enrichment results mainly involved in the following four aspects: (i) inflammation-associated activities, such as regulation of reactive oxygen species metabolic process, reactive oxygen species biosynthetic process, and cellular response to oxidative stress; (ii) cell cycle, such as negative regulation of apoptotic signaling pathway and negative regulation of extrinsic apoptotic signaling pathway; (iii) angiogenesis, such as regulation of blood vessel endothelial cell migration; and (iv) physiological process, such as female gonad development and mammary gland development.

### 3.6. KEGG Pathway Analysis

The KEGG enrichment analysis of 68 target genes was performed using R software. We finally got a total of 146 items including the 44 key signaling pathways listed in Table 3. We conducted network visualization via Cytoscape as plotted in Figure 3(d).

### 3.7. Molecular Docking Analysis

Among 28 core targets, the top 5 target proteins in terms of degree were chosen for molecular docking, including AKT1, TNF, SRC, CASP3, and JUN, which were considered the key targets in the process of KP treating SOP. To verify how KP binds to the key targets, we adopted molecular docking using AutoDock Vina to predict their docking interactions.  Table 4 shows the docking results including affinity and interaction information.

**Figure 4:**
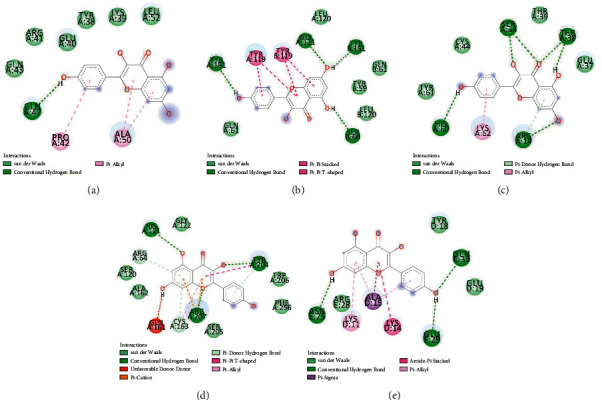
Simulated molecular docking of kaempferol on AKT1 (a), TNF (b), SRC (c), CASP3 (d), and JUN (e).

According to Figure 4(a), KP combined with AKT1 by forming one hydrogen bond with the residue Gln-47 and six van der Waals interactions with Gln-43, Arg-41, Glu-40, Tyr-38, Lys-39, and Leu-52 (binding affinity: −6.0 kcal/mol). In addition, there were pi-alkyl interactions upon KP with Pro-42 and Ala-50.

According to Figure 4(b), the combination affinity of KP on TNF was −7.6 kcal/mol. The residues containing Leu-120, Gln-61, and Tyr-59 interacted with KP by forming 5 van der Waals interactions. Moreover, KP combined with TNF by forming four hydrogen bonds with the residues Gly-121, Ser-60, and Tyr-151. Notably, there were pi-pi stacked and pi-pi T-shaped interactions between KP and Tyr-119.

According to Figure 4(c), the combination affinity of KP on SRC was −5.9 kcal/mol. There existed pi-alkyl interaction and pi-donor hydrogen bond, respectively, provided by the Lys-62 and Arg-14 residues in the interactions with KP. Moreover, KP was bound with the residues Ser-36, Thr-38, Arg-14, and His-60 by hydrogen bonds and Glu-37, Thr-39, Cys-44, and Tyr-61 by van der Waals.

According to Figure 4(d), the combination affinity of KP on CASP3 was −8.4 kcal/mol. There were 6 van der Waals interactions provided by the Gly-122, Ser-120, Ala-162, Ser-205, Phe-256, and Trp-206 residues in the interactions with KP. There existed pi-alkyl interaction and pi-donor hydrogen bonds provided by the Cys-163, Tyr-204, and Arg-64 residues in the interactions with KP. Additionally, KP combined with CASP3 by forming three hydrogen bonds with the residues Arg-207, His-121, and Tyr-204 and an unfavorable donor-donor interaction with Gln-161. Notably, there were pi-cation and pi-pi T-shaped interactions upon KP with Arg-207 and Tyr-204.

According to Figure 4(e), the combination affinity of KP on JUN was −6.4 kcal/mol. There existed 3 hydrogen bonds provided by the Asn-25, Glu-29, and Gln-33 residues in the interactions with KP. Additionally, there were 3 van der Waals interactions upon KP with Arg-28, Tyr-18, and Glu-19. Notably, KP interacted with the Lys-11, Lys-14, and Ala-15 residues by pi-sigma, pi-alkyl, and amide-pi stacked interactions.

## 4. Discussion

KP, a flavonoid identified in *Drynariae Rhizoma*, has been revealed to have beneficial effects on SOP via inhibiting osteoclast formation and bone loss [12, 20]. Studies have illustrated that KP exerts the antiosteoporotic function via upregulating microRNA-101 and activating the Wnt/*β*-catenin pathway, which promotes osteoblast differentiation, proliferation, and migration [21]. To further explore the mechanisms of KP in treating SOP, we carried out a series of bioinformatics analysis to screen potential targets and pathways in the present study.

In our present study, we got 68 overlapped targets between KP and SOP, including 28 core targets listed in Table 2. According to PPI network topology analysis, we noticed that these targets were characteristics of inflammation, oxidative stress, and bone homeostasis-associated proteins. The top five targets ranked by degree are AKT1, TNF, SRC, CASP3, and JUN, which are all bound tightly with KP according to molecular docking results, indicating that they may play a key role in KP treatment for SOP.

AKT1 (RAC-alpha serine/threonine-protein kinase) is identified as a unique signaling intermediate in bone homeostasis that controls the differentiation of osteoblasts and osteoclasts [22]. Some studies have verified that the inhibition of AKT1 expression would enhance bone turnover markers' expression and extracellular matrix mineralization, which consequently suppresses osteoporosis [23]. Moreover, AKT1 plays an important role in the PI3K-Akt signaling pathway, the involvement of which alleviates SOP progression by suppressing inflammatory response and osteoclast formation [24]. Moreover, evidence shows that kaempferol could block AKT1 phosphorylation [25]. Therefore, we speculated that KP could reduce inflammatory response and osteoclast formation by downregulating AKT1 expression levels in patients suffering from SOP, thus exerting therapeutic effects on SOP.

TNF (tumor necrosis factor) is the earliest inflammatory mediator produced in response to oxidative stress and promotes the production of inflammatory mediators and induces the expression of macrophage colony-stimulating factor (M-CSF) [26]. TNF affects SOP healing by activating NF-*к*B, promoting RANKL-induced osteoclast differentiation, and increasing bone resorption [27]. TNF-*α* plays a critical role in the development of osteoporosis via regulating oxidative stress, bone homeostasis, and remodeling [28, 29]. Moreover, the existing study reveals that KP could significantly decrease the TNF expression and secretion [30]. Therefore, we speculated that KP could reduce oxidative stress in inflammatory response by downregulating TNF expression in SOP patients, so as to anti-SOP.

SRC (Proto-oncogene tyrosine-protein kinase Src) has been reported to involve in the process of osteoblast differentiation, which plays a vital role in advancing bone maturation [31]. Further studies have revealed that SRC plays a pivotal role in driving osteoblast proliferation and extracellular matrix (ECM) remodeling, which influences bone formation and remodeling [32]. Moreover, SRC is also an osteoclast-specific gene, which is essential for osteoclast function [33]. In general, the involvement of SRC exerts important effects on bone metabolism, which participates in the regulation of osteoblast and osteoclast activities [34]. Notably, sufficient evidence has revealed that KP regulates anti-inflammatory responses by the direct suppression of SRC [35]. However, research is needful to explore whether KP could exert therapeutic effects on SOP by regulating the expression of SRC and thus suppressing inflammatory response.

CASP3 (caspase-3) gets involved in cell apoptosis [36]. Evidence has revealed that the downregulation of CASP3 mRNA can promote SOP healing [37]. Further studies have demonstrated that the upregulation of CASP3 can activate the p53 signaling pathway, destroy osteoblast maturation, and inhibit chondrocyte differentiation, thus restraining SOP healing [38]. It has been verified that CASP3 deletion could alleviate inflammatory response [39]. Moreover, it has been reported that KP treatment could remarkably decrease the CASP3 expression *in vitro* [40]. Nevertheless, there are scanty research projects exploring the regulation of KP on CASP3 expression to alleviate inflammatory response for treating SOP.

JUN is a proinflammatory factor and forms a dimer complex called AP-1 along with FOS, which accelerates the transcription and expression of genes related to bone growth and development containing AP-1 binding sites through multiple mechanisms, thus regulating bone metabolism [41, 42]. Numerous studies have confirmed that the activation of JUN promotes osteoclastogenesis [43]. Moreover, JUN, as a regulatory factor in the JNK signaling pathway, could activate inflammatory response and osteoclast formation [44]. Notably, sufficient evidence has revealed that KP regulates anti-inflammatory responses by the suppression of JUN [45, 46]. Thus, JUN plays a key role in inflammatory response and osteoclastogenesis and KP might treat SOP by suppressing JUN expression.

Similar to PPI analysis, GO enrichment results show consistent results. Additionally, biological processes involving the regulation of inflammatory response, oxidative stress, and bone homeostasis make a key role in KP treating SOP, as demonstrated in Figure 3(b). In recent years, reports have revealed that inflammatory response plays an important role in the pathogenesis of SOP, which could disrupt bone homeostasis by accelerating bone resorption and inhibiting bone formation, thereby triggering SOP [47]. Mounting evidence reveals the role of KP in attenuating inflammatory response by encumbering the expressions of inflammatory mediators in many signaling pathways like MAPK [48]. For example, the MAPK signaling pathway being activated would promote the expressions of inflammatory cytokines TNF-*α* and IL-1*β* in inflammatory response, while the presence of KP could suppress this pathway and exert protective effects on SOP [7, 49]. KP, modulating the activities of proinflammatory enzyme, has been reported to inhibit cyclooxygenase expression in numerous inflammatory disorders [50]. KP also suppresses the production of nitric oxide that triggers the activation of TNF-*α*, thereby inhibiting inflammatory response [51]. There is growing evidence for the role of kaempferol in attenuating inflammatory response mediated by NF-*к*B, indicating its protective effects on bone loss in postmenopausal osteoporosis by blocking TNF-*α*-induced nuclear translocation of the NF-*к*B subunit p65 from the cytoplasm to the nucleus [52]. And KP could suppress age-related NF-*к*B activation by inhibition of NF-*к*B subunit p65 translocation so as to restrain inflammatory response [53]. Some evidence showed that there was a negative correlation between dietary intake level of KP and serum CRP level, suggesting the key role of KP in reducing the risk of inflammation [54]. It has been verified that oxidative stress makes key functions in SOP-related inflammatory response [55]. Oxidative stress can alter bone homeostasis, accelerate bone resorption, and reduce bone formation, leading to the progression of SOP [56]. And some evidence has illustrated the therapeutic effects of kaempferol on the damage induced by oxidative stress and inflammation in osteoporosis [7, 57], suggesting that kaempferol is a natural antioxidant for treating osteoporosis. According to our present study, kaempferol may be an antioxidant with a good prospect that helps reduce inflammatory response and oxidative stress, thus improving SOP. Moreover, accumulating studies have revealed that the expressions of core targets, including AKT1 [58], TNF [29], SRC [35], CASP3 [59], JUN [44], etc., make vital functions in regulating inflammatory response and oxidative stress. Therefore, we speculated that KP could regulate core targets' expressions and bone homeostasis by inflammatory response and oxidative stress in SOP patients, so as to anti-SOP.

KEGG enrichment results revealed that PI3K-Akt, MAPK, VEGF, prolactin, HIF-1, TNF, estrogen, IL-17, p53, and NF-kappa B (NF-*к*B) signaling pathways may exert regulatory functions on kaempferol against SOP.

The involvement of PI3K-Akt signaling pathway alleviates SOP progression by suppressing inflammatory response and osteoclast formation [24]. Moreover, some studies have shown that the PI3K-Akt signaling pathway is involved in the inhibition of osteoporosis through promoting osteoblast proliferation, differentiation, and bone formation [60, 61], Therefore, the PI3K-AKT signaling pathway is essential in bone homeostasis.

Inflammatory pathways including IL-17 [62], TNF [63], and NF-*к*B [64] signaling pathways participate in regulating osteoclast differentiation. Moreover, the IL-17 signaling pathway can stimulate the synthesis of TNF-*α*, IL-6, and NF-*к*B, thereby promoting RANKL-induced osteoclast differentiation [65]. Therefore, IL-17, TNF, and NF-*к*B signaling pathways are speculated to exert important functions in the process of KP treatment against SOP, which needs further identification.

The estrogen signaling pathway can exert regulatory functions on osteoblasts' and osteoclasts' proliferation, apoptosis, and differentiation [66]. The current study has revealed that KP regulates osteoblastic differentiation via estrogen receptor signaling [67]. Similar to the estrogen signaling pathway, research on the prolactin signaling pathway also indicates that KP has the function of estrogen regulation, which is evidence for kaempferol in treating postmenopausal SOP [68].

Evidence has confirmed that the activation of p53 signaling pathway can disrupt osteoblast maturation and restrain chondrocyte differentiation [38]. The inhibition of MAPK signaling pathway suppresses osteoclastogenesis [69]. The activation of HIF-1/VEGF signaling pathway can accelerate angiogenesis in bone tissues, which gets involved in the pathological evolution of SOP [70, 71].

In summary, our results predict some potential therapeutic targets and pathways, providing reference for future studies on KP treatment against SOP. However, one limitation of this study is that further *in vivo* and *in vitro* experiments are needed to confirm our findings.

## 5. Conclusion

Collectively, our results first reveal that KP may treat SOP possibly via regulating inflammatory response, oxidative stress, bone homeostasis, etc. These results will provide theoretical basis for KP treatment against SOP. However, the specific mechanism and material basis still need to be further verified *in vivo* and *in vitro*.

## Figures and Tables

**Table 1:** Potential target genes of KP in the treatment of SOP.

Number	Gene
1	NOS2
2	PTGS1
3	AR
4	PPARG
5	PTGS2
6	HSP90AA1
7	PIK3CG
8	PRKACA
9	DPP4
10	PGR
11	F2
12	NOS3
13	RELA
14	AKT1
15	BCL2
16	BAX
17	TNF
18	JUN
19	CASP3
20	MAPK8
21	XDH
22	MMP1
23	STAT1
24	HMOX1
25	CYP3A4
26	CYP1A1
27	ICAM1
28	VCAM1
29	ALOX5
30	AHR
31	INSR
32	GSTM1
33	SLPI
34	NOX4
35	AKR1B1
36	TYR
37	CA2
38	ABCB1
39	GLO1
40	SYK
41	GSK3B
42	MMP9
43	MMP2
44	CDK5
45	CCNB1
46	ESR2
47	TTR
48	CYP19A1
49	EGFR
50	IGF1R
51	MPO
52	PIK3R1
53	CA1
54	SRC
55	PTK2
56	KDR
57	MMP13
58	MMP3
59	MET
60	BACE1
61	AKR1A1
62	APP
63	PARP1
64	MMP12
65	ESR1
66	CFTR
67	TERT
68	MAPT

**Table 2:** Core targets of KP in the treatment of SOP.

Number	Core targets	Degree
1	TNF	56
2	AKT1	52
3	SRC	47
4	CASP3	45
5	JUN	45
6	EGFR	43
7	PTGS2	43
8	HSP90AA1	41
9	MMP9	41
10	ESR1	39
11	PPARG	39
12	MAPK8	32
13	MMP2	32
14	NOS3	30
15	RELA	29
16	HMOX1	28
17	GSK3B	28
18	KDR	26
19	AR	26
20	ICAM1	26
21	IGF1R	26
22	STAT1	25
23	VCAM1	25
24	PGR	24
25	PTK2	24
26	APP	22
27	PIK3R1	22
28	MPO	21

**Table 3:** KEGG pathway enrichment analysis.

ID	Signaling pathway	Enriched gene number	*p* value
hsa04933	AGE-RAGE signaling pathway	15	1.25*E* − 15
hsa04926	Relaxin signaling pathway	14	1.24*E* − 12
hsa04915	Estrogen signaling pathway	14	3.18*E* − 12
hsa04657	IL-17 signaling pathway	12	8.40*E* − 12
hsa04668	TNF signaling pathway	12	6.95*E* − 11
hsa04917	Prolactin signaling pathway	9	3.91*E* − 09
hsa04625	C-type lectin receptor signaling pathway	10	8.98*E* − 09
hsa04066	HIF-1 signaling pathway	10	1.42*E* − 08
hsa04151	PI3K-Akt signaling pathway	15	1.04*E* − 07
hsa04012	ErbB signaling pathway	8	3.64*E* − 07
hsa04370	VEGF signaling pathway	7	4.16*E* − 07
hsa04010	MAPK signaling pathway	13	5.19*E* − 07
hsa04211	Longevity regulating pathway	8	5.21*E* − 07
hsa04064	NF-kappa B signaling pathway	8	1.73*E* − 06
hsa04071	Sphingolipid signaling pathway	8	4.78*E* − 06
hsa04722	Neurotrophin signaling pathway	8	4.78*E* − 06
hsa04014	Ras signaling pathway	10	1.56*E* − 05
hsa04664	Fc epsilon RI signaling pathway	6	1.70*E* − 05
hsa04620	Toll-like receptor signaling pathway	7	1.94*E* − 05
hsa04660	T cell receptor signaling pathway	7	1.94*E* − 05
hsa04062	Chemokine signaling pathway	9	2.23*E* − 05
hsa04662	B cell receptor signaling pathway	6	4.96*E* − 05
hsa04919	Thyroid hormone signaling pathway	7	5.18*E* − 05
hsa05235	PD-L1 expression and PD-1 checkpoint pathway	6	7.88*E* − 05
hsa04068	FoxO signaling pathway	7	8.60*E* − 05
hsa05022	Pathways of neurodegeneration	13	9.65*E* − 05
hsa04912	GnRH signaling pathway	6	0.000100782
hsa04213	Longevity regulating pathway	5	0.000138239
hsa04072	Phospholipase D signaling pathway	7	0.000184632
hsa04921	Oxytocin signaling pathway	7	0.000236022
hsa04015	Rap1 signaling pathway	8	0.000278775
hsa04152	AMPK signaling pathway	6	0.000406963
hsa04621	NOD-like receptor signaling pathway	7	0.000693065
hsa04910	Insulin signaling pathway	6	0.000822176
hsa04150	mTOR signaling pathway	6	0.001558955
hsa04024	cAMP signaling pathway	7	0.002012705
hsa04920	Adipocytokine signaling pathway	4	0.002323
hsa04115	p53 signaling pathway	4	0.002854606
hsa04371	Apelin signaling pathway	5	0.005314536
hsa04630	JAK-STAT signaling pathway	5	0.010013151
hsa04340	Hedgehog signaling pathway	3	0.010486159
hsa04020	Calcium signaling pathway	6	0.01301751
hsa04622	RIG-I-like receptor signaling pathway	3	0.019110112
hsa04550	Signaling pathways regulating pluripotency of stem cells	4	0.028737678

**Table 4:** Molecular interactions of key targets and KP.

Compound	Target	PDB ID	Affinity (kcal/mol)	Interactions
Kaempferol	AKT1	1UNQ	−6.0	Hydrogen bond, pi-alkyl, van der Waals
Kaempferol	TNF	2AZ5	−7.6	Hydrogen bond, van der Waals, pi-pi stacked, pi-pi T-shaped
Kaempferol	SRC	1O41	−5.9	Hydrogen bond, pi-alkyl, van der Waals
Kaempferol	CASP3	1NMS	−8.4	Hydrogen bond, pi-alkyl, pi-pi T-shaped, pi-cation, unfavorable donor-donor, van der Waals
Kaempferol	JUN	5FV8	−6.4	Hydrogen bond, pi-alkyl, pi-sigma, van der Waals, amide-pi stacked

## Data Availability

The data sets used and analyzed during the current study are available from the first author on reasonable request.

## Supplementary Materials

Supplementary Materials Supplementary Table S1: the structure of kaempferol. Supplementary Table S2: targets of kaempferol.
