# Supplementary material for: Research on the Mechanism of Kaempferol for Treating Senile Osteoporosis by Network Pharmacology and Molecular Docking
Source: Evid Based Complement Alternat Med. 2022 Feb 3;2022:6741995. doi: 10.1155/2022/6741995 (PMC8831051; doi:10.1155/2022/6741995)
Supplement: Supplementary Materials — Supplementary Table S1: the structure of kaempferol. Supplementary Table S2: targets of kaempferol. [file 6741995.f1.docx]

**Supplementary Table S1. The Structure of Kaempferol**

| Molecule ID | PubChem CID | Molecule name | Chemical formula | Structure | OB(%) | DL |
| --- | --- | --- | --- | --- | --- | --- |
| MOL000422 | 5280863 | Kaempferol | C_15_H_10_O_6_ | 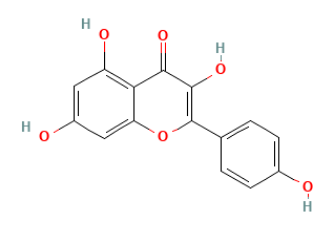 | 41.88 | 0.24 |

OB: oral bioavailability; DL: drug-likeness

**Supplementary Table S2. Targets of Kaempferol**

| Number | Gene symbol | Number | Gene symbol | Number | Gene symbol |
| --- | --- | --- | --- | --- | --- |
| 1 | NOS2 | 52 | INSR | 103 | CSNK2A1 |
| 2 | PTGS1 | 53 | DIO1 | 104 | EGFR |
| 3 | AR | 54 | PPP3CA | 105 | AVPR2 |
| 4 | PPARG | 55 | GSTM1 | 106 | IGF1R |
| 5 | PTGS2 | 56 | GSTM2 | 107 | PIM1 |
| 6 | HSP90AA1 | 57 | AKR1C3 | 108 | AURKB |
| 7 | PIK3CG | 58 | SLPI | 109 | DRD4 |
| 8 | PRKACA | 59 | NOX4 | 110 | MPO |
| 9 | NCOA2 | 60 | AKR1B1 | 111 | PIK3R1 |
| 10 | DPP4 | 61 | TYR | 112 | PYGL |
| 11 | PRSS1 | 62 | FLT3 | 113 | CA1 |
| 12 | PGR | 63 | CA2 | 114 | SRC |
| 13 | F2 | 64 | CA7 | 115 | PTK2 |
| 14 | CHRM1 | 65 | HSD17B2 | 116 | KDR |
| 15 | NOS3 | 66 | ABCC1 | 117 | MMP13 |
| 16 | GABRA2 | 67 | HSD17B1 | 118 | MMP3 |
| 17 | ACHE | 68 | CA12 | 119 | CA3 |
| 18 | SLC6A2 | 69 | ESRRA | 120 | PLK1 |
| 19 | CHRM2 | 70 | ABCB1 | 121 | CA6 |
| 20 | ADRA1B | 71 | ABCG2 | 122 | PKN1 |
| 21 | GABRA1 | 72 | ADORA1 | 123 | CA14 |
| 22 | TOP2A | 73 | CA4 | 124 | CA9 |
| 23 | F7 | 74 | MAOA | 125 | MET |
| 24 | CALM1 | 75 | GLO1 | 126 | NEK2 |
| 25 | RELA | 76 | SYK | 127 | CXCR1 |
| 26 | IKBKB | 77 | GSK3B | 128 | CAMK2B |
| 27 | AKT1 | 78 | MMP9 | 129 | ALK |
| 28 | BCL2 | 79 | MMP2 | 130 | NEK6 |
| 29 | BAX | 80 | ALOX15 | 131 | PLA2G1B |
| 30 | TNF | 81 | ALOX12 | 132 | CA5A |
| 31 | JUN | 82 | PTPRS | 133 | BACE1 |
| 32 | AHSA1 | 83 | ADORA2A | 134 | AXL |
| 33 | CASP3 | 84 | CDK5R1 | 135 | NUAK1 |
| 34 | MAPK8 | 85 | CDK5 | 136 | AKR1C2 |
| 35 | XDH | 86 | CCNB3 | 137 | AKR1C1 |
| 36 | MMP1 | 87 | CDK1 | 138 | AKR1C4 |
| 37 | STAT1 | 88 | CCNB1 | 139 | CA13 |
| 38 | HMOX1 | 89 | CCNB2 | 140 | AKR1A1 |
| 39 | CYP3A4 | 90 | ARG1 | 141 | APP |
| 40 | CYP1A1 | 91 | GPR35 | 142 | PARP1 |
| 41 | ICAM1 | 92 | ESR2 | 143 | MMP12 |
| 42 | SELE | 93 | DAPK1 | 144 | CD38 |
| 43 | VCAM1 | 94 | MPG | 145 | TOP1 |
| 44 | NR1I2 | 95 | SLC22A12 | 146 | ESR1 |
| 45 | CYP1B1 | 96 | TTR | 147 | CFTR |
| 46 | ALOX5 | 97 | AKR1B10 | 148 | PFKFB3 |
| 47 | HAS2 | 98 | TNKS2 | 149 | AMY1A |
| 48 | AHR | 99 | TNKS | 150 | GRK6 |
| 49 | PSMD3 | 100 | CDK6 | 151 | TERT |
| 50 | SLC2A4 | 101 | CDK2 | 152 | MAPT |
| 51 | NR1I3 | 102 | CYP19A1 |  |  |
